# Exploring the impact of a data-feedback workshop on injury trends in a regional collegiate American football league in Japan

**DOI:** 10.3389/fspor.2026.1746737

**Published:** 2026-04-30

**Authors:** Yasuyuki Umezaki, Yasuhiro Endo, Naoki Taguchi, Toshiya Urushihata, Hiroki Kataoka, Kousei Hiratsuka, Hiroshi Katou, Yoshimi Akaihata, Noriaki Maeda, Hiroshi Shinohara

**Affiliations:** 1 Graduate School of Health Sciences, Aomori University of Health and Welfare, Aomori, Japan; 2 Department of Physical Therapy, Fukushima Medical University, Fukushima, Japan; 3 Faculty of Sports Science, Sendai University, Miyagi, Japan; 4 Department of Physical Therapy, Sendai Medical and Sports College, Miyagi, Japan; 5 Department of Physical Therapy, M&Medical Reha Co., Ltd., Yamagata, Japan; 6 Tohoku Collegiate American Football Association, Miyagi, Japan; 7 Faculty of Sport Sciences, Waseda University, Saitama, Japan

**Keywords:** collegiate football, data feedback, educational intervention, injury prevention, Tohoku region

## Abstract

The accurate ascertainment of injury epidemiology within a target population is the first step in sports injury prevention. However, in regional collegiate American football leagues in Japan, collected surveillance data have not been adequately disseminated back to the teams and players. This mixed-methods study, comprising a cross-sectional survey and a pre-post ecological analysis, aimed to explore the impact of integrating league-specific injury surveillance data into a mandatory workshop for team representatives. A non-randomized, uncontrolled pre-post study was conducted in the Tohoku Collegiate American Football League in Japan. Injury data from 2018 to 2023 served as the baseline, and the analytical findings were presented as feedback during the 2024 workshop. We compared participants’ perceptions through pre- and post-intervention questionnaires, and evaluated official injury reports from the single 2024 season (post-intervention). No severe injuries were reported during either period. Following the workshop, self-reported safety awareness among participants improved. The overall injury rate per game did not significantly change (Incidence Rate Ratio: 1.22, 95% CI: 0.70–2.18). However, observed injury proportions shifted: muscle cramps decreased [37.2% (*n* = 145) vs. 7.7% (*n* = 3), *p* < 0.05], while head injuries increased [6.2% (*n* = 24) vs. 15.4% (*n* = 6), *p* < 0.05]. Data-driven feedback was associated with heightened awareness and a proportional decrease in readily addressable issues, such as muscle cramps. The proportional increase in head injuries may reflect improved reporting awareness rather than an absolute risk increase. Given the absence of a control group and the small post-intervention sample size, these exploratory findings suggest that this approach is a practical initial step for resource-limited organizations, warranting further controlled studies.

## 1 Introduction

American football is characterized by a high incidence of various injuries due to its nature as a contact-intensive sport. These include concussions, other head and neck injuries, lower limb ligament damage, and heat-related illnesses (1, 2). As these injuries can affect not only an athlete's career longevity but also their long-term health and quality of life (3, 4), the establishment of effective preventive measures in sports settings is a critical issue. A widely referenced framework for injury prevention is the Sequence of Prevention model by van Mechelen et al. (5). This model advocates for constructing prevention strategies through a cyclical process: 1) ascertaining the extent of the injury problem, 2) identifying risk factors, 3) implementing preventive measures, and 4) evaluating the effectiveness of the intervention. While diverse preventive strategies exist, including rule changes and equipment improvements (6–8), educational interventions for athletes and staff are considered an important strategy for enhancing safety awareness and potentially facilitating sustainable safety practices on the field.

Educational interventions, such as workshops on safety management in sports, are widely implemented to enhance player safety awareness (9). In Japan, where many athletes begin their American football careers at the university level, the thorough dissemination of fundamental knowledge to prevent severe incidents like concussions and heatstroke is indispensable. The Tohoku Collegiate American Football League has also conducted a mandatory annual workshop, which has contributed to the reinforcement of safety measures throughout the league. However, standard educational interventions often rely solely on generalized knowledge transfer. To effectively translate injury prevention strategies into real-world sports settings, the Translating Research into Injury Prevention Practice (TRIPP) framework emphasizes the crucial role of the implementation context, such as the limited resources and specific environments of regional leagues (10). In line with this implementation science perspective, recent injury surveillance literature highlights the necessity of utilizing data collected from specific teams and leagues. This localized approach enables players and staff to make informed decisions tailored to their own circumstances (11, 12). Consequently, despite the demand for more practical, data-driven educational intervention methods, there has been a scarcity of research in implementation science that directly provides feedback using specific injury surveillance data from the players’ own league and verifies its impact.

Therefore, this study utilized the existing framework of the Tohoku Collegiate American Football League's workshop. We modified the conventional lecture-based format by analyzing the league's previously unutilized long-term injury data and subsequently feeding back the specific injury risks faced by the league to its members. The purpose of this study was to explore whether utilizing local injury data in a mandatory safety workshop influences participant awareness and subsequent injury outcomes. The specific research questions were: (1) Does the integration of league-specific injury data into the workshop increase self-reported knowledge, awareness, and intent to implement safety practices among players and staff (Phase I)? and (2) Is this data-driven educational intervention associated with a reduction in the overall game injury rate or a shift in the proportional patterns of injuries (Phase II)? This study is anticipated to provide practical insights into the design and operation of injury prevention strategies in regional leagues, serving as an exploratory case study for evaluating a data-driven feedback cycle that links surveillance with educational intervention in resource-limited settings.

## 2 Materials and methods

### 2.1 Study design

This research employed a non-randomized, uncontrolled, pre-post observational design. It combined a cross-sectional questionnaire study of participants evaluating the workshop (Phase I) with an ecological pre-post comparison of game-related injury incidence trends (Phase II).

### 2.2 Participants and data sources

This study was conducted within the Tohoku Collegiate American Football League, comprising eight teams.

For Phase I (the questionnaire survey), the target population consisted of all registered individuals for the 2024 season: 176 players and 79 student staff members (total: 255). To distribute the survey, the URL was sent to the chief administrators of each team, who were instructed to share it with their respective players and staff. Because this was an indirect distribution, we could not confirm the exact number of individuals who received the link, making the calculated response rates approximate. Ultimately, 43 valid responses (approximate response rate: 16.9%; 27 players, 16 staff) for the pre-survey and 22 valid responses (approximate response rate: 8.6%; 13 players, 9 staff) for the post-survey were analyzed.

For Phase II, we analyzed official injury reports from autumn season games (2018–2024). These reports were systematically recorded during games by game physicians. This injury report form is widely recognized as a standard tool for injury surveillance in Japanese collegiate American football (13). Although the reports were digitized via Google Forms, the limited administrative resources of this regional league required several months post-season for the league office to aggregate and verify the data for research purposes. Consequently, access to these retrospective data was performed as follows: data for the pre-intervention period (2018–2023) were accessed from June 7, 2024, to July 21, 2024, and data for the post-intervention period (2024 season) were accessed from July 22, 2025, to August 25, 2025. This study was approved by the Research Ethics Committee of Aomori University of Health and Welfare (Approval Nos. 24015, 24020). For the questionnaire survey, a thorough explanation was provided to all participants, both orally and in writing, before obtaining their informed consent. Due to the retrospective nature of the injury data analysis, information about the research was posted on the websites of the affiliated institution and related organizations in lieu of individual written or oral explanations.

### 2.3 Phase I: data-feedback-driven mandatory workshop

At the end of July 2024, prior to the autumn season, a 60-minute online mandatory workshop was conducted. Each team was required to have at least two representatives, such as the captain and medical staff, attend the session. These representatives were subsequently tasked with disseminating the information to their teammates.

The core content of this workshop was initially designed in response to the league's specific request to fulfill the educational needs for the prevention and initial management of concussions and heatstroke. To maximize educational impact within the practical constraints of the 60-minute timeframe, the session included standard emergency procedures, such as cardiopulmonary resuscitation (CPR), automated external defibrillator (AED) usage, Emergency Action Plan (EAP) preparation, and basic heatstroke and concussion protocols. In addition to this essential baseline education, as the unique approach of this study, the analytical results of the league's own past 6-year injury trends (2018–2023) were incorporated into the presentation. This data-driven feedback specifically highlighted local risks, such as the high prevalence of muscle cramps and a recent proportional increase in head/neck injuries, while connecting these findings to practical preventive strategies (e.g., hydration and proper tackling techniques). This integrated approach aimed to visualize the league's specific challenges and stimulate practical safety awareness beyond generic knowledge transfer.

### 2.4 Survey design and questionnaire

The questionnaire survey was conducted anonymously using Google Forms, and participation was on a voluntary basis. It consisted of two independent cross-sectional surveys: a pre-survey assessing baseline conditions and a post-survey evaluating the workshop's impact. Most items utilized a 4-point Likert scale, with responses scored from 1 to 4 for quantitative analysis.

To capture relevant constructs, the pre-survey included specific sample questions such as: “To what extent is your knowledge about specific injuries such as calf cramps and head/neck injuries?” (Knowledge); “To what extent are you concerned about safety measures during practice?” (Awareness/Concern); and “To what extent do you focus on proper tackling form during regular practice?” (Practice). The post-survey evaluated changes and future intentions with questions such as: “After attending the seminar, has your awareness regarding injuries increased?” (Awareness change), and included an open-ended question: “Specifically, what do you plan to do in practice starting tomorrow?” (Plan/Intention). The content of the free-text responses was subjected to qualitative thematic analysis.

The questionnaire was originally developed by the authors to reflect the specific context of the league's injury landscape. To enhance the content validity, it was supervised by a research advisor specializing in sports rehabilitation who is a certified athletic trainer of the Japan Sport Association. All questionnaire items used are presented in Supplementary Material S1. While this expert review provided content validity, formal psychometric validation, such as reliability testing, was not conducted, which is a recognized limitation of this phase.

### 2.5 Phase II: comparison of injury incidence trends

To evaluate injury trends, we analyzed official game reports. Injuries occurring during practices were excluded because standardized reports were only mandated during games. In this study, an injury was defined as any event during an official game that prompted a referee-called timeout, resulting in the player's temporary removal from the field (2). Unlike the widely used “time-loss” injury definition (which requires a player to miss at least one day of participation), this referee-timeout-based definition captures immediate game-day incidents regardless of subsequent days lost. While this approach effectively standardizes recording during games with limited medical staff, it may underestimate minor injuries that do not stop the play, or conversely, capture transient issues (e.g., mild cramps) where the player quickly returns to the same game. Data extracted included the total number of injuries, the average number of injuries per game, injury types, injured body parts, specific diagnoses, player position, game quarter, academic year, and type of play. The data from the post-intervention year (2024) were compared with the six-year pre-intervention period (2018–2023). Furthermore, we also monitored the occurrence of severe cases. A severe case was defined in this study, in accordance with the Japan American Football Association's criteria for reporting serious incidents (14), as an event that was life-threatening or had the potential to result in significant long-term sequelae (e.g., death, intracranial hemorrhage, severe neck injuries, internal organ damage, severe heatstroke, or cardiac arrest).

### 2.6 Statistical analysis

Statistical analyses were performed using R version 4.2.1 (CRAN, freeware), and the significance level was set at 5%. For the Likert-scale questionnaire responses, the Mann–Whitney U test was employed for intergroup comparisons.

The Game Injury Rate per 1000 Athlete-Exposures (GIR) was calculated. Given the limited administrative capacity of the regional league to accurately record the exact number of players exposed per play or per game, we estimated athlete-exposures using the total number of registered players multiplied by the number of games. This practical estimation method aligns with recent epidemiological approaches validated in major Japanese collegiate American football leagues (2).

To compare the mean number of injuries per game between Period a (2018–2023) and Period b (2024), we initially considered a Poisson regression model. However, testing revealed significant overdispersion (the residual deviance substantially exceeded the degrees of freedom). Therefore, a Negative Binomial regression model with the log of the number of games as an offset term was utilized to calculate the Incidence Rate Ratio (IRR) and 95% confidence interval (CI). Categorical proportions were compared using the *χ*^2^ test or Fisher's exact test. For items where the *χ*^2^ test revealed a statistically significant difference, adjusted residual analysis was conducted as a *post-hoc* test. A significant deviation was determined if the absolute value of the adjusted residual was greater than 1.96. Given the exploratory nature of this study and the small post-intervention sample size, adjustments for multiple comparisons were not applied. Thus, changes in proportional distribution should be interpreted cautiously as observed trends rather than definitive causal effects.

## 3 Results

### 3.1 Comprehension of the mandatory workshop (Phase I)

The respondents for the questionnaire survey, administered to evaluate the workshop, comprised 43 participants (player group: *n* = 27; team staff group: *n* = 16) for the pre-survey and 22 participants (player group: *n* = 13; team staff group: *n* = 9) for the post-survey.

#### 3.1.1 Pre-survey

The primary results of the pre-survey (*n* = 43), which served as a baseline assessment prior to the workshop, are presented in Table 1. Overall, both players and team staff demonstrated moderate levels of knowledge regarding general and specific injuries. However, the results of the Mann–Whitney U test revealed that the team staff group reported a statistically significantly higher score on the item “concern about future injuries” compared to the player group (*p* = 0.02). Other items concerning safety awareness and practice did not show significant differences between the two groups at baseline.

**Table 1:** Comparison of injury perceptions between players and team staff before the mandatory workshop.

Question Item	Total (*n* = 43)	Players (*n* = 27)	Team Staff (*n* = 16)	*p*-value
Questions on Knowledge and Awareness				
Q1. Knowledge of injuries	3 [2–3]	3 [2–3]	3 [2–3.25]	0.4
Q2. Knowledge of specific injuries	2 [2–3]	2 [2–3]	3 [2–3]	0.4
Q4. Concern for safety measures	3 [3–4]	3 [3–4]	4 [3–4]	0.06
Questions on Practice				
Q6. Focus on proper tackling form	3 [3–4]	3 [3–4]	3 [2.75–4]	0.4
Q9. Concern about future injuries	3 [3–4]	3 [3–3]	3.5 [3–4]	0.02*

Data are expressed as Median [Interquartile Range].

*p*-value; Team Staff vs. Players (Mann–Whitney U test).

This table presents the results for the main question items representing the categories of “Knowledge,” “Awareness,” “Practice,” and “Concern.” All question items are shown in Supporting information S2-1 and S2-2.

#### 3.1.2 Post-survey

**Figure 1:**
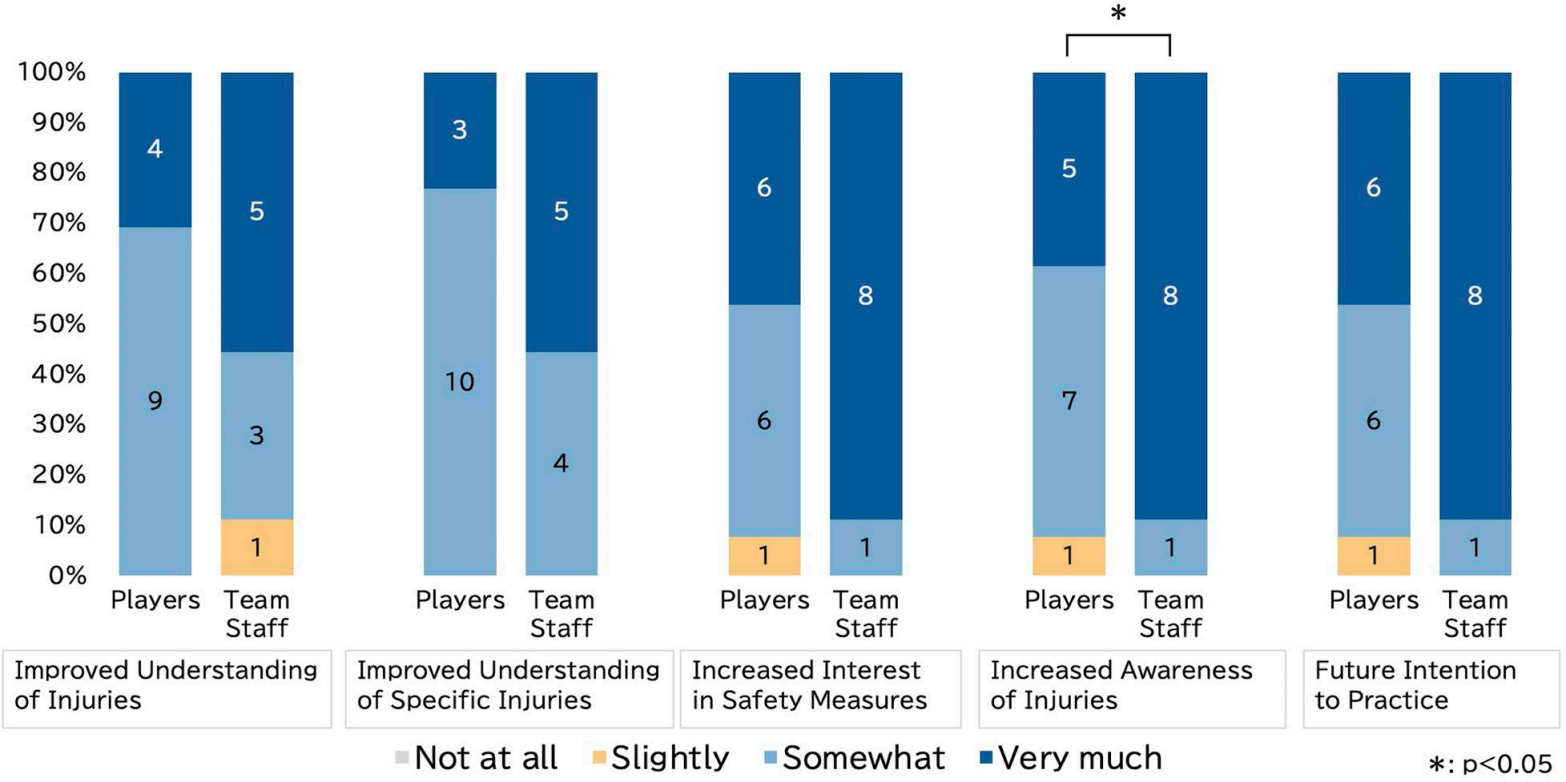
Group comparison of post-workshop questionnaire responses. Responses to each item were categorized into four rating levels (Level 4: Very much, Level 3: Somewhat, Level 2: Slightly, Level 1: Not at all), and the proportions are presented as a 100% stacked bar chart. The specific wording of the response options varied depending on the question (e.g., “deepened,” “increased,” “thought”). For a full list, see Supplementary materials S2-1, S2-2.

The results of the post-survey (*n* = 22), conducted immediately following the workshop, are presented in Figure 1. While the overall post-workshop evaluations were highly positive across all participants, the team staff group demonstrated a statistically significantly higher score than the player group on the questionnaire item regarding ‘enhanced awareness of injuries’ [Median (Interquartile Range): 4 (4–4) for team staff vs. 3(3–4) for players; *p* < 0.05]. Specifically, the proportion of respondents who selected ‘greatly enhanced’ was 88.9% (*n* = 8/9) in the team staff group, whereas it was 38.5% (*n* = 5/13) in the player group.

For the remaining items, no statistically significant differences were observed between the groups, with both groups reporting high median scores [e.g., for ‘increased interest in safety measures’, median scores were 4 (4–4) for team staff and 3 (3–4) for players]. Furthermore, for all items, the combined proportion of participants who responded ’strongly agree’ or ’somewhat agree’ exceeded 90%. In the open-ended question asking, ’Specifically, what do you plan to do?’, a variety of qualitative responses reflecting the workshop's data-feedback content were provided. These responses were subjected to qualitative thematic analysis and categorized based on content similarity (detailed in Supplementary S8). The primary categories identified included “Heatstroke Prevention and Hydration”, “Injury Prevention”, “Emergency Response Preparedness,” and “Observation and Communication.” Notably, several responses were directly related to the prevention of muscle cramps, including intentions such as ‘to hydrate regularly given that cramps were reported to be frequent’ and ‘to encourage frequent hydration’.

### 3.2 Pre- and post-intervention injury incidence trends (Phase II)

To explore the potential impact of the mandatory workshop on injury incidence trends, data from the pre-intervention period (Period a: 2018–2023) and the post-intervention period (Period b: 2024) were compared. It should be noted that in the 2024 season, the total number of games included in the analysis was limited to six, as several games were canceled due to team-specific circumstances such as the occurrence of COVID-19 cases and existing injuries. Consequently, the total number of injuries in Period b was small (*n* = 39), which requires caution when interpreting proportional changes. Throughout both the pre- and post-intervention periods, there were zero reported cases of severe injuries.

#### 3.2.1 Overall injury rates

**Figure 2:**
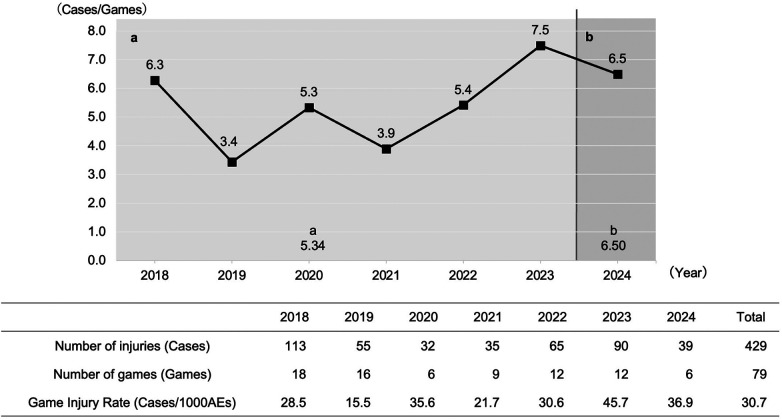
Temporal trends in the average number of injuries per game and the Game Injury Rate per 1000 Athlete-Exposures in the Tohoku Collegiate American Football League. The annual trends in the average number of injuries per game and the Game Injury Rate per 1000 Athlete-Exposures (GIR) are presented. The GIR was calculated by dividing the total number of injuries in each year by the total athlete-exposures (number of registered players × number of games) and multiplying by 1000 {GIR = [Total injuries/(Registered players × Games)] × 1000}. Period a (2018–2023) represents the pre-intervention period, and Period b (2024) represents the post-intervention period.

During the six-year pre-intervention period (Period a), 390 injuries occurred over 73 games (average 5.34 injuries per game). In the post-intervention year (Period b), 39 injuries occurred over 6 games (average 6.50 injuries per game) (Figure 2). A Negative Binomial regression analysis, using the log of the number of games as an offset term to account for overdispersion, was performed to compare the per-game injury rate. The results indicated no statistically significant difference in the injury incidence rate post-intervention compared to the pre-intervention period (IRR: 1.22, 95% CI: 0.70–2.18, *p* = 0.488). The average Game Injury Rate (GIR) for all injuries over the seven years of official games was 30.7 per 1000 athlete-exposures.

#### 3.2.2 Changes in injury proportions

**Figure 3:**
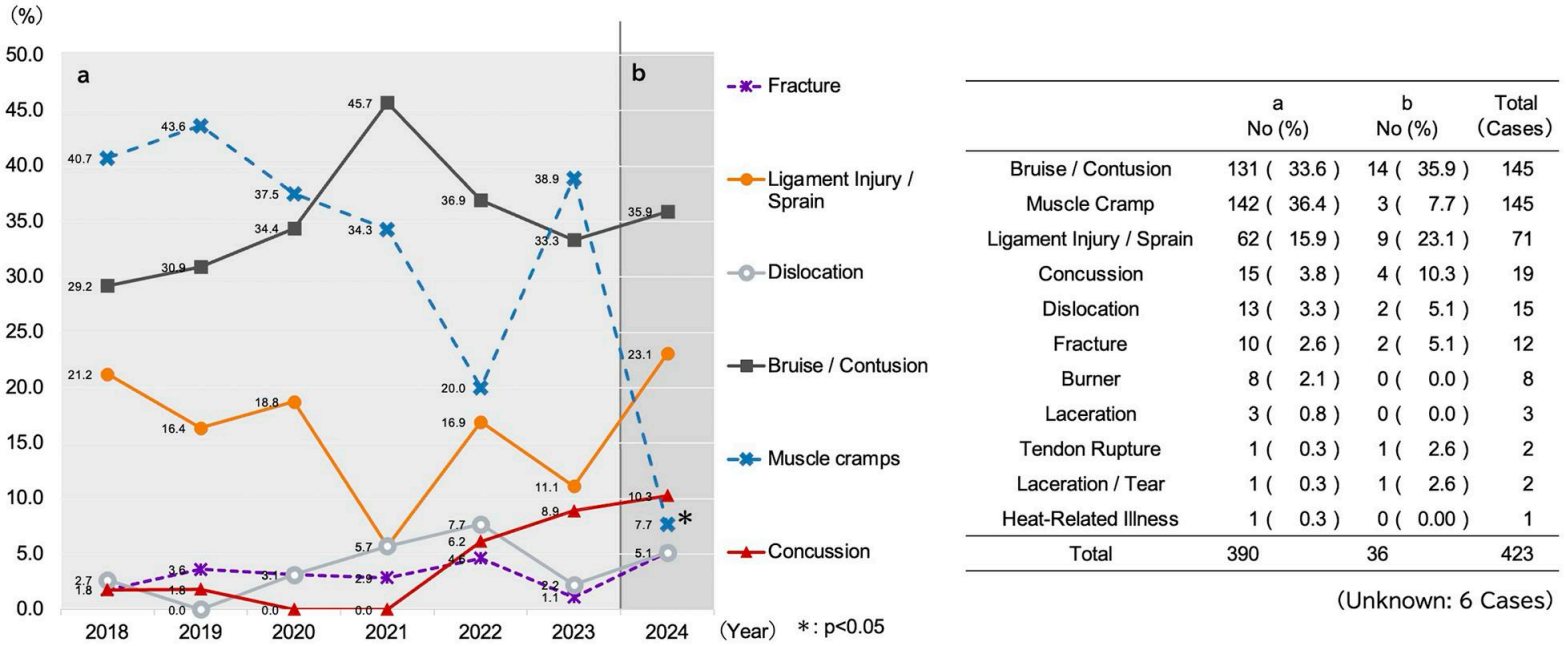
Temporal trends in the proportion of injury types. This figure displays a line graph (left) illustrating the annual trends in the proportion (%) of the top six injury types among all recorded injuries, with a corresponding data table (right). Period a (2018–2023) represents the pre-intervention period, and Period b (2024) represents the post-intervention period. *; *p* < 0.05 vs. Period a (Adjusted residual analysis following *χ*^2^ test).

Figures 3 through 5 display the time-series data for injury characteristics. The symbols (*) within the figures indicate the periods in which the proportion of occurrence was found to be statistically significantly different from Period a, as determined by adjusted residual analysis following a *χ*^2^ test.

**Figure 4:**
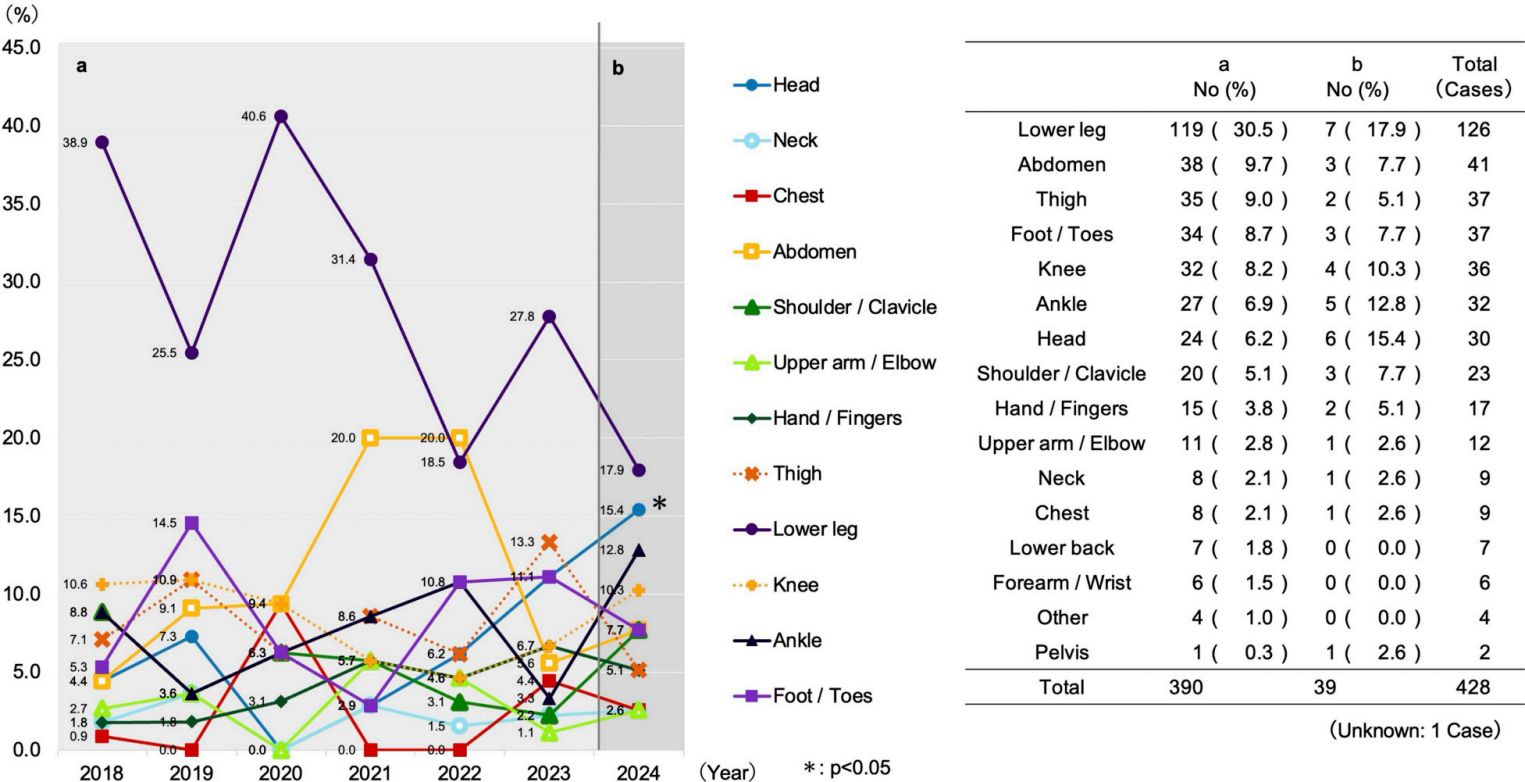
Temporal trends in the proportion of injured body parts. This figure displays a line graph (left) illustrating the annual trends in the proportion (%) of injured body parts among all recorded injuries, with a corresponding data table (right). Period a (2018–2023) represents the pre-intervention period, and Period b (2024) represents the post-intervention period. *; Adjusted residual analysis following *χ*^2^ test (*p* < 0.05).

**Figure 5:**
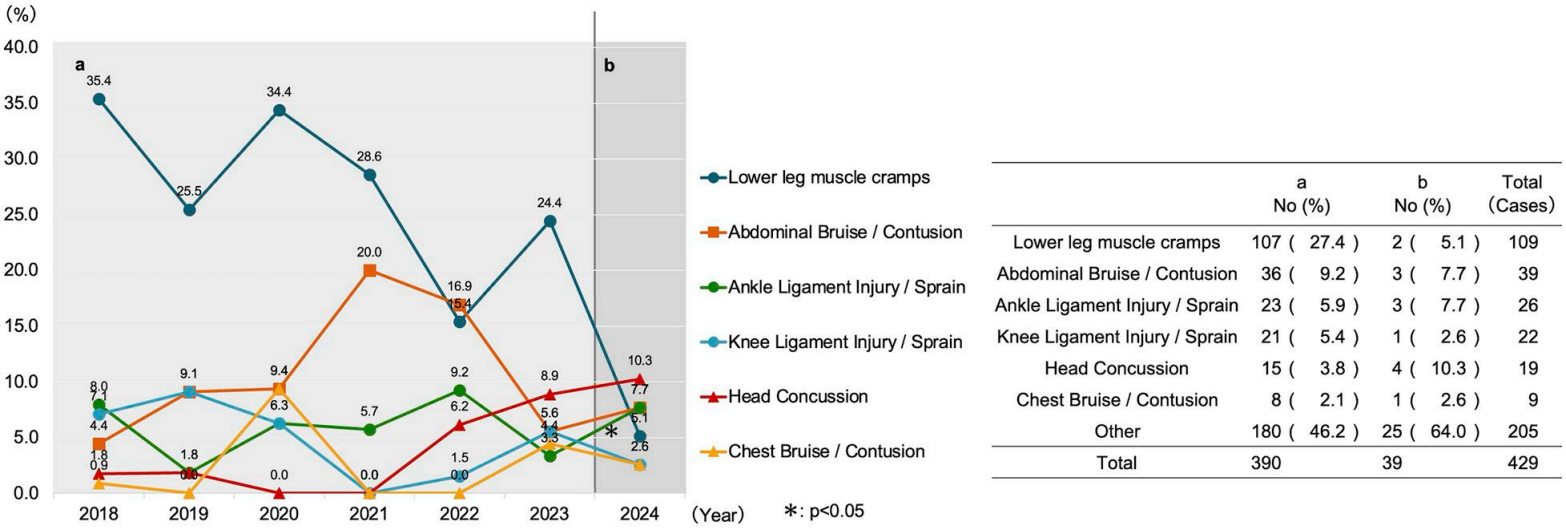
Temporal trends in the proportion of major injury diagnoses. This figure displays a line graph (left) illustrating the annual trends in the proportion (%) of major injury diagnoses among all recorded injuries, with a corresponding data table (right). Period a (2018–2023) represents the pre-intervention period, and Period b (2024) represents the post-intervention period. *; Adjusted residual analysis following *χ*^2^ test (*p* < 0.05).

A comparison of the injury breakdown revealed significant proportional shifts. By injury type, the proportion of muscle cramps significantly decreased from 37.2% (*n* = 145/390) pre-intervention to 7.7% (*n* = 3/39) post-intervention (*p* < 0.05) (Figure 3). By injured body part, the proportion of head injuries significantly increased from 6.2% (*n* = 24/390) pre-intervention to 15.4% (*n* = 6/39) post-intervention (*p* < 0.05) (Figure 4). By specific injury diagnosis, lower leg muscle cramps showed a significant decrease (Figure 5). A detailed cross-tabulation of all injury diagnoses and body parts is provided in Supplementary Material S3.

#### 3.2.3 Other variables

No statistically significant changes were observed between the pre- and post-intervention periods for other categorical variables, including player position, game quarter, academic year, and type of play. The detailed temporal trends and proportion tables for these variables have been provided in the Supplementary Materials (Figures S4–S7).

## 4 Discussion

This study explored the potential impact of feeding back league-specific injury data during a mandatory safety workshop. While the overall injury rate per game did not change significantly post-intervention, the pattern of injury types shifted. Given the observational design and the small post-intervention sample size, these findings should be interpreted cautiously as exploratory observations rather than definitive causal effects.

The observed decrease in the proportion of muscle cramps suggests the intervention may have enhanced awareness regarding easily addressable issues like hydration, which participants frequently mentioned in the post-survey. In contrast to top-tier Japanese collegiate leagues where ligament injuries are predominant (2, 15, 16), regional leagues frequently report muscle cramps as a major challenge, often linked to structural limitations in conditioning support (17–20). However, muscle cramps are heavily influenced by unmeasured confounding factors, including annual weather variations, preseason training loads, and team conditioning levels. Therefore, this reduction likely resulted from an interplay of these variables rather than the workshop alone.

Conversely, the proportional increase in head injuries requires a dual interpretation: an actual increase in true risk exposure vs. improved reporting awareness. Although objective kinematic data were not collected, field observations suggest that factors prevalent in regional leagues, including varying levels of player experience and constraints within overall coaching and training environments, can lead to inadequate tackling skills. Previous studies have highlighted that players are particularly vulnerable to head impacts during periods of unacclimated physical load, such as the preseason (11). A 60-minute lecture is inherently insufficient to modify complex motor skills, potentially leaving players exposed to high-impact risks during games. Alternatively, the education provided may have fostered a proactive safety culture, leading players and staff to report minor head impacts that were previously ignored. This suggests that the observed rise in head injuries could reflect improved detection and reporting bias rather than an absolute increase in risk exposure. Indeed, increased concussion knowledge has been significantly associated with higher reporting rates of minor head impacts among student-athletes (21, 22). Both factors underscore that data-feedback must be coupled with practical, on-field skills training to effectively mitigate head injury risks.

From an implementation science perspective, this study serves as a practical application of the Audit and Feedback strategy in a resource-limited sports setting (12). Recent implementation research in sports injury prevention emphasises that translating interventions into real-world practice is heavily impacted by local contexts, coach motivation, and limited resources (23). Providing specific local data helped visualize the league's structural challenges and navigate these barriers. For similar regional leagues, utilizing existing mandatory meetings to disseminate injury data offers a feasible first step in building a continuous prevention cycle. Future research should employ cluster-randomized controlled trials comparing data-feedback combined with practical skills training against standard education alone. Furthermore, while this study successfully observed longitudinal changes in injury trends at the league level, the underlying differences among individual universities in their attitudes and specific approaches to injury prevention remain unexamined. Therefore, future research must integrate qualitative interview studies targeting players and team staff. Exploring these team-specific safety cultures and practical barriers to implementation will complement the epidemiological surveillance data by explaining the contextual mechanisms behind the observed statistical trends, ultimately informing the development of more tailored and effective preventive strategies.

## 5 Limitations

This study has several substantial limitations. First, the non-randomized, uncontrolled pre-post design precludes causal inference; we cannot definitively determine whether the workshop caused the observed changes in injury proportions. Second, the post-intervention period was limited to a single season comprising only six games and a total of 39 injuries. This extremely low injury count renders percentage estimates unstable, limits statistical power, and makes it difficult to distinguish true trends from random variation. Third, there are critical limitations regarding the Phase I survey: the sample sizes were small, the approximate response rates were low (likely due to the indirect distribution method), the participants differed between the pre- and post-surveys, and the custom-developed questionnaire lacked formal psychometric validation for reliability. These factors significantly weaken the robustness of the awareness assessment and raise the possibility of selection and social desirability biases. Fourth, various unquantified confounding variables, including weather conditions, technical skill levels, rule-related factors, and reporting awareness, were not adjusted for in the statistical models. Consequently, given that this study focused exclusively on a single regional collegiate league in Japan, the generalizability of these findings to other populations or competitive levels is limited.

## 6 Conclusions

Integrating local injury data into a mandatory safety workshop served as a practical tool for visualizing the unique, multifactorial challenges faced by a regional collegiate American football league. Following this exploratory data-feedback approach, we observed improved self-reported awareness alongside a proportional decrease in readily manageable issues like muscle cramps. However, this process also highlighted the limitations of brief educational interventions in addressing complex risks requiring technical skills, such as head injuries. For resource-limited sports organizations, this data-driven feedback cycle offers a feasible initial framework to stimulate a proactive safety culture.

## Data Availability

The original contributions presented in the study are included in the article/Supplementary Material, further inquiries can be directed to the corresponding author.
